# CPAP pressure and flow data at 2 positive pressure levels and multiple controlled breathing rates from a trial of 30 adults

**DOI:** 10.1186/s13104-022-06133-w

**Published:** 2022-07-16

**Authors:** Ella F. S. Guy, Jennifer L. Knopp, Oliver Gilbertson, Simon Blue, Lui Holder-Pearson, J. Geoffrey Chase

**Affiliations:** Department of Mechanical Engineering, Centre for Bioengineering, University of Canterbury, Christchurch, New Zealand

**Keywords:** Biomedical Engineering, Pulmonary mechanics, Respiratory mechanics, Respiratory modelling, CPAP, PEEP

## Abstract

**Objectives:**

A unique dataset of airway flow/pressure from healthy subjects on Continuous Positive Airway Pressure (CPAP) ventilation was collected. This data can be used to develop or validate models of pulmonary mechanics, and/or to develop methods to identify patient-specific parameters which cannot be measured non-invasively, during CPAP therapy. These models and values, particularly if available breath-to-breath in real-time, could assist clinicians in the prescription or optimisation of CPAP therapy, including optimising PEEP settings.

**Data description:**

Data was obtained from 30 subjects for model-based identification of patient-specific lung mechanics using a specially designed venturi sensor system comprising an array of differential and gauge pressure sensors. Relevant medical information was collected using a questionnaire, including: sex; age; weight; height; smoking history; and history of asthma. Subjects were tasked with breathing at five different rates (including passive), matched to an online pacing sound and video, at two different levels of PEEP (4 and 7 cmH_2_O) for between 50 and 180 s. Each data set comprises ~ 17 breaths of data, including rest periods between breathing rates and CPAP levels.

## Objective

CPAP is a form of non-invasive ventilation used in both hospital and home settings to treat respiratory conditions and wean patients off invasive ventilation [1, 2]. Positive end-expiratory pressure (PEEP) is the key CPAP setting. Insufficient PEEP can result in under-oxygenation or airway collapse [3, 4]. Conversely, excessive PEEP causes pulmonary barotrauma [3, 4]. Rupture of tissue (emphysema) and air trapping can lead to pneumothorax [3, 4]. Excessive PEEP can also cause increases in intracranial pressure due to intrathoracic pressure increases, as well as increased fluid retention [3, 5]. Hence, the setting is critical care and outcomes, as well as carrying risk with sub-optimal settings.

Protocols for PEEP settings vary significantly and are predominantly based on clinical judgment of the comparative foreseeable risks of hyperinflation and under oxygenation [6–8]. Thus, variability of results and inequity of care can lead to issues of unconscious bias [9]. In CPAP ventilation, the “CPAP Titration Protocol” [6, 10, 11] is commonly used to titrate PEEP based on patient blood oxygenation or symptoms of airway collapse.

The described dataset [12] has been used to develop a method of extrapolating patient breathing effort and ventilator unloading non-invasively in CPAP therapy [13]. Objective, model-based quantification of ventilator unloading in CPAP creates novel real-time monitoring. This metric provides further, novel feedback to clinicians on the efficacy of the CPAP therapy at given PEEP settings with the potential to guide clinical decision support and care. More specifically, the goal of CPAP is to support the work of breathing, so a metric quantifying this support in real-time would offer potential clinical value. Future work will involve a larger trial with a secondary measurement method to further validate the model using a larger subject pool.

## Data description

Data was collected using a customised sensor system consisting of a Venturi tube on either side of the CPAP expiration hole (Fig. 1). The dual-Venturi apparatus (Fig. 2) with a central expiration hole was 3D printed to fit standard CPAP masks (FreeMotion RT041, Fisher and Paykel Healthcare, New Zealand), with an inner diameter of 15 mm (D1) and a venturi diameter of 12 mm (D2). The data set [12] thus captures flow delivered from the CPAP machine and flow delivered to the CPAP mask.

Differential pressure sensors connected in both directions over the Venturi restrictions enable flow measurement (Fig. 3), and static pressure was also measured at both locations. As flow sensors were uni-directional, both inspiratory and expiratory flow were measured at each Venturi tube separately, using two sets of differential pressure (flow) sensors.

Data was recorded at a frequency of 83.33 Hz (resampled to 100 Hz) from the sensors via a central Arduino unit (Nano V3, Baite Electronics, China) in analogue-to-digital converter (ADC) counts, which was serially interfaced with a laptop for data acquisition. The sensor system design approach is open access with details in [14]. Sensors were calibrated against a known flow profile obtained from a hospital-grade mechanical ventilator (PB840, Puritan Bennet, USA, using CURESoft [15]).

The trial protocol consisted of a sequence of breaths at resting, 6, 9, 30, and 60 breaths per minute, repeated at nominal PEEP levels of 4 and 7 cmH_2_O. At each cued breathing rate, 17 breaths were cued for inhalation and exhalation by an audio-visual pacer.

Raw data was processed in Matlab (Matlab 2020a, The Mathworks Inc, Natick, MA, USA), with pressure values calculated based on datasheet information for each of the sensors [16, 17]. The flow was calculated from differential pressure (∆P = P_1− _P_2_) across each Venturi as a function of its relationship to the ratio decrease in cross-sectional tube area at the venturi restriction ($\frac{{\mathrm{A}}_{1}}{{\mathrm{A}}_{2}}$) by derivation of Bernoulli and continuity equations (Eq. 1), yielding:

1 $$ {\text{Q}} = {\text{c}}_{{\text{d}}} {\text{A}}_{2} \sqrt {\frac{{2\left( {\Delta {\text{P}}} \right)}}{{{\uprho }(1 - \left( {\frac{{{\text{A}}_{1} }}{{{\text{A}}_{2} }}} \right)^{2} }}} $$

Given a drag coefficient (c_d_) of 0.97 and the density of air (ρ) as 1.225 kg/m^3^, and assuming total flow into the Venturi restriction equals the total flow out of the same Venturi.

The inspiratory and expiratory flow were merged into a single multidirectional flow dataset for each Venturi by a minimum inspiration length and volume based on the cued breath rate. The final data set thus has flow in [L/s] and pressure in [cmH_2_O] at two locations: at the entry to the CPAP mask (Q_1_, P_2_) and downstream of the expiration hole (Q_2_, P_2_) as illustrated in Fig. 1.

Data (Table 1) [12] is collated into Excel files and organised into raw (“RAW_CSV_Data_Files”) and processed data (“Processed_CSV_Data_Files”) folders. Both Folders contain subfolders of the 10 PEEP and breath rate combinations, which in turn contain an excel file per subject. Raw data is arranged in columns of time, pressure, and flow (at both venturis and in both directions). Processed data is arranged in columns of time, pressure, and flow (at both venturis). Corresponding subject demographic data is provided as an excel file organised in columns by subject, sex, age, weight, height, BMI, smoking history, and vaping history. A “README” text document is also included outlining how the data a stored.

**Table 1:** Overview of data files/data sets

Label	Name of data file/data set	File types (file extension)	Data repository and identifier (DOI or accession number)
Data set 1	Processed_CSV_Data_Files	.zip	Physionet https://doi.org/10.13026/xfae-vv63 [12]
Data set 2	RAW_CSV_Data_File	.zip	Physionet https://doi.org/10.13026/xfae-vv63 [12]
Data file 1	README.txt	.txt	Physionet https://doi.org/10.13026/xfae-vv63 [12]
Data file 2	SubjectDemographicData.csv	.csv	Physionet https://doi.org/10.13026/xfae-vv63 [12]
Data file 3	Figure 1.png	.png	Physionet https://doi.org/10.13026/xfae-vv63 [12]
Data file 4	Figure 2.png	.png	Physionet https://doi.org/10.13026/xfae-vv63 [12]
Data file 5	Figure 3.png	.png	Physionet https://doi.org/10.13026/xfae-vv63 [12]
Data file 6	LICENSE.txt	.txt	Physionet https://doi.org/10.13026/xfae-vv63 [12]
Data file 7	SHA256SUMS.txt	.txt	Physionet https://doi.org/10.13026/xfae-vv63 [12]

## Limitations

Data with significant observed sensor error was removed during processing. Significant error was considered when no distinguishable breaths (inspiration and expiration) were captured. These breaths showed no flow from the two differential pressure sensors. A potential source of this error was moisture in the pressure tubing causing pressure sensor failure for some smaller breaths due to moisture blocking the sensor tube. The dataset remains unedited, and these breaths were simply not processed. These breaths comprised less than 20% of the data in the entire data set. 2% of the data in the set was not processed due to human error in recording trial data. A more optimally fitted mask would be expected to reduce the error in fully capturing expiration, by reducing leaks around the mask’s seal with the face.

The inclusion of subjects with significant respiratory abnormalities and/or induced respiratory distress would provide more comprehensive data for the development of clinical metrics, decision-support systems, and/or clinical CPAP protocols. The preliminary patient WOB and ventilator unloading findings [13] establish the value of this data, and hence an extended trial is scheduled to collect information from a larger and more diverse (in age, ethnicity, and respiratory condition) subject pool.

## Data Availability

The datasets generated and/or analysed during the current study are available on Physionet [12].

## Abbreviations

ARDS: Acute respiratory distress syndrome
CPAP: Continuous positive airway pressure
E: Elastance
P: Pressure
PEEP: Positive end expiratory pressure
Q: Flow
WOB: Work of breathing
